# Make Acetylcholine Great Again! Australian Skinks Evolved Multiple Neurotoxin-Proof Nicotinic Acetylcholine Receptors in Defiance of Snake Venom

**DOI:** 10.3390/ijms26157510

**Published:** 2025-08-04

**Authors:** Uthpala Chandrasekara, Marco Mancuso, Glenn Shea, Lee Jones, Jacek Kwiatkowski, Dane Trembath, Abhinandan Chowdhury, Terry Bertozzi, Michael G. Gardner, Conrad J. Hoskin, Christina N. Zdenek, Bryan G. Fry

**Affiliations:** 1 Adaptive Biotoxicology Lab, School of the Environment, University of Queensland, St Lucia, QLD 4072, Australia; uthpalachandrasekara@yahoo.com (U.C.); 19marcomancuso19@gmail.com (M.M.); lee.jones1@student.uq.edu.au (L.J.); abhichy.official@gmail.com (A.C.); 2 Department of Biological, Geological and Environmental Sciences, University of Catania, Via Etnea, 64, 95131 Catania, CT, Italy; 3 Sydney School of Veterinary Science, B01, University of Sydney, Sydney, NSW 2006, Australia; glenn.shea@sydney.edu.au; 4 Australian Museum Research Institute, The Australian Museum, Sydney, NSW 2010, Australia; dane.trembath@australian.museum; 5 Queensland Emory Drug Discovery Initiative, Uniquest, University of Queensland, St Lucia, QLD 4072, Australia; j.kwiatkowski@uniquest.com.au; 6 South Australian Museum, North Terrace, Adelaide, SA 5000, Australia; terry.bertozzi@samuseum.sa.gov.au; 7 School of Biological Sciences, The University of Adelaide, Adelaide, SA 5001, Australia; 8 College of Science and Engineering, Flinders University, GPO Box 2100, Adelaide, SA 5001, Australia; michael.gardner@flinders.edu.au; 9 College of Science & Engineering, James Cook University, Townsville, QLD 4811, Australia; conrad.hoskin@jcu.edu.au; 10 Australian Reptile Academy, Ripley, QLD 4306, Australia; christinazdenek@gmail.com

**Keywords:** venom, evolution, adaptation, nicotinic acetylcholine receptor, neurotoxin, skink

## Abstract

Many vertebrates have evolved resistance to snake venom as a result of coevolutionary chemical arms races. In Australian skinks (family Scincidae), who often encounter venomous elapid snakes, the frequency, diversity, and molecular basis of venom resistance have been unexplored. This study investigated the evolution of neurotoxin resistance in Australian skinks, focusing on mutations in the muscle nicotinic acetylcholine receptor (nAChR) α1 subunit’s orthosteric site that prevent pathophysiological binding by α-neurotoxins. We sampled a broad taxonomic range of Australian skinks and sequenced the nAChR α1 subunit gene. Key resistance-conferring mutations at the toxin-binding site (N-glycosylation motifs, proline substitutions, arginine insertions, changes in the electrochemical state of the receptor, and novel cysteines) were identified and mapped onto the skink organismal phylogeny. Comparisons with other venom-resistant taxa (amphibians, mammals, and reptiles) were performed, and structural modelling and binding assays were used to evaluate the impact of these mutations. Multiple independent origins of α-neurotoxin resistance were found across diverse skink lineages. Thirteen lineages evolved at least one resistance motif and twelve additional motifs evolved within these lineages, for a total of twenty-five times of α-neurotoxic venoms resistance. These changes sterically or electrostatically inhibit neurotoxin binding. Convergent mutations at the orthosteric site include the introduction of N-linked glycosylation sites previously known from animals as diverse as cobras and mongooses. However, an arginine (R) substitution at position 187 was also shown to have evolved on multiple occasions in Australian skinks, a modification previously shown to be responsible for the Honey Badger’s iconic resistance to cobra venom. Functional testing confirmed this mode of resistance in skinks. Our findings reveal that venom resistance has evolved extensively and convergently in Australian skinks through repeated molecular adaptations of the nAChR in response to the enormous selection pressure exerted by elapid snakes subsequent to their arrival and continent-wide dispersal in Australia. These toxicological findings highlight a remarkable example of convergent evolution across vertebrates and provide insight into the adaptive significance of toxin resistance in snake–lizard ecological interactions.

## 1. Introduction

Venom functions as a critical ecological adaptation shaped by predator–prey dynamics, promoting diversification within venomous systems [1]. This evolutionary arms race drives increased toxin specificity for physiological targets, enhancing prey susceptibility and aiding in their capture [2]. In turn, prey and predators of venomous animals have evolved defensive adaptations that confer resistance, mitigating the impact of venom through various strategies, such as altering toxin–target interactions to decrease binding affinity [3,4,5,6,7,8,9]. This coevolutionary interplay is propelled by mutual selective pressures exerted by venom components and prey defences, exemplifying a perpetual evolutionary race consistent with the Red Queen Hypothesis [10,11,12,13]. Consequently, these persistent evolutionary interactions foster ongoing phenotypic and genotypic diversification among predator and prey species [14,15].

As a result, prey species have not merely survived but flourished in environments populated by venomous predators, highlighting the powerful influence of natural selection within the predator–prey evolutionary arms race [12,16]. A classic example of this is the predator–prey venom innovation–venom resistance chemical arms race between rattlesnakes and squirrels [11,12,17,18,19,20,21,22,23]. This ongoing adaptive competition exemplifies the remarkable resilience and adaptability organisms exhibit when facing severe survival threats. Exploring this evolutionary dynamic provides valuable insights into fundamental natural selection processes and the delicate balance maintained within ecosystems, where predators and prey continually coevolve, shaping each other’s defensive and offensive adaptations. Resistance mechanisms vary widely among species [9], with two particularly critical strategies identified: toxin-neutralisation via circulating proteins that bind toxins before they reach their intended physiological targets [17,24,25], and structural alterations in the toxin targets themselves, reducing toxin binding affinity [3,5,6,7,8]. As resistance has been noted to be secondarily lost in some lineages subsequent to radiating out into ecological niches devoid of alpha-neurotoxic venomous snakes, this suggests that resistance comes with a fitness disadvantage imposed by less efficient or slower binding by the neurotransmitter acetylcholine against the structurally changed receptor [26,27].

Venomous snakes impose strong selection pressure upon their prey (and also predators). In neurotoxic snakes, a key component is three-finger toxins (α-neurotoxins), which bind to the alpha-1 subunit of the skeletal muscle nicotinic acetylcholine receptor (nAChR) at neuromuscular junctions, causing paralysis and death [28,29,30]. These snake venom postsynaptic neurotoxins may target the orthosteric (acetylcholine-binding) site of the muscle nAChR α1 subunit, a receptor critical for neuromuscular signalling, or target allosteric sites that do not competitively inhibit binding by acetylcholine to the receptor but bind to other sites on the receptor and impede function [28,29,30,31,32,33,34,35]. While the sites of allosteric binding by snake venom α-neurotoxins are poorly characterised, in contrast, orthosteric binding is well-defined with two main binding sites known for snake venom α-neurotoxins: an aromatic binding site, which includes amino acid positions 187 and 189; and the proline site, which includes the prolines at positions 194 and 197 [36]. These interactions may be highly prey selective [2,32,34,37,38,39,40,41,42,43,44], such as sea snake venoms being most potent on fish [32,44] and king cobra venoms being most potent on snakes [2,44]. In response to this selection pressure, some prey and predators of venomous snakes have evolved resistance, sparking an evolutionary arms race. All the currently documented forms of venom resistance that have been described to-date are due to changes in the orthosteric site of the alpha-1 subunit [3,4,9,26,27,36,45,46,47,48,49,50,51,52,53,54,55,56,57,58]. Small changes in this receptor’s binding site can prevent toxin binding without disrupting normal acetylcholine function. Two main mechanisms have evolved to confer resistance: steric hindrance and electrostatic repulsion.

Steric hindrance is the physical impedance of the ability of a toxin to bind through changes in the three-dimensional architecture of the orthosteric site, and this is accomplished through two primary mechanisms: N-glycosylation of an asparagine [45,56,57,58] or substitution of a proline [36]. The addition of an N-glycosylation sequon (N-X-S/T, where N is asparagine and X is any amino acid except cysteine (C) or proline (P), followed by S = serine or T = threonine) [59,60,61] near the orthosteric site leads to the post-translational attachment of a bulky carbohydrate moiety, which physically obstructs the docking of snake venom α-neurotoxins. This glycan acts like a molecular shield, blocking the neurotoxin from accessing key receptor residues required for high-affinity binding—while leaving acetylcholine signalling largely unaffected due to differences in molecular size and binding dynamics. This is the most efficient form of resistance described to-date [36]. N-glycosylation was first documented in the mongoose, conferring resistance to the venom of its cobra prey [45]. Subsequently, it was shown to be a basal trait within the Herpestidae family (mongooses and meerkats) [27,51]. This mutation was also shown to be the mechanism by which elapid snakes avoid self-intoxication, and is a basal trait within these snakes [27,57,58]. Further studies showed N-glycosylation evolving convergently in a myriad of elapid snake prey species, including caecilian amphibians, agamid lizards, and viperid snakes [27,47,55]. Similarly, proline substitutions at structurally flexible positions in binding loop regions (such as loops B or C) can disrupt the local secondary structure of the receptor, introducing kinks or rigidity that deform the toxin-binding site. Therefore, their loss (whether through substitution or codon deletion) would change the three-dimensional geometry of the orthosteric site. These conformational changes can prevent the neurotoxin from achieving the precise molecular fit required for effective inhibition, thus reducing or eliminating its paralytic effects. Proline substitution has also been documented in diverse predators or prey of elapid snakes, including caecilians and varanid lizards (which may be either predator or prey depending on the respective sizes on either side of the interaction) [26,55].

The electrostatic repulsion mechanism of α-neurotoxin resistance is achieved through the substitution of positively charged amino acids at critical positions within the orthosteric site of the nAChR α1 subunit. Snake venom α-neurotoxins possess a strong net positive charge, which guides the initial binding to the negatively charged surface of the receptor’s ligand-binding domain [2]. However, when a positively charged residue is introduced at a key binding-loop position, it creates electrostatic interference that repels the cationic neurotoxin. This repulsion disrupts the toxin’s ability to dock stably onto the receptor, thereby preserving normal cholinergic function and preventing venom-induced paralysis. Importantly, this resistance strategy has evolved convergently in several lineages, highlighting the predictability and efficacy of electrostatic repulsion as a biochemical defence against neurotoxic venoms. This introduction of a positively charged amino acid arginine (R) at position 187 was hypothesised [50] and subsequently experimentally demonstrated [52] to be the single mutation responsible for the iconic resistance of the Honey Badger (*Mellivora capensis*) to its cobra prey. Another form of electrostatic charge repulsion, in which the ancestral negatively charged amino acids aspartic acid (D) or glutamic acid (E) at positions 191 or 195 were replaced by the positively charged amino acid lysine (K), was shown to have evolved in slow-moving terrestrial Afro–Asian snakes that were vulnerable to the abundant sympatric cobras (*Naja* species) and king cobras (*Ophiophagus* species) [46,52]. It has also been shown that the elimination of both negatively charged amino acids from the orthosteric site (191 and 195 simultaneously), even without the replacement with positively charged amino acids, is enough to lessen the binding affinity of α-neurotoxins to the receptor, thereby conferring a level of venom resistance (but significantly lower than the introduction of positive charges at these sites) [26,54].

Other forms of resistance involve complicated interactions of amino acid sites that are not easily definable to single types of changes, unlike the steric hindrance and electrostatic charge repulsion mutations described above. Afro–Asian primates sympatric with cobras are, in general, less susceptible to snake venom neurotoxins than primates who do not coexist with these large diurnal snakes. This resistance is at its most potentiated in the Homininae clade, which consists of the bipedal lineages. The Homininae resistance was shown to be due to a combination of serine (S) introduced at position 187 in place of tryptophan (W) and phenylalanine (F) in place of threonine (T) at position 189, with both mutations needing to be in place to confer resistance [52].

While venom resistance is well documented in a myriad of animals, smaller lizards like skinks have not been systematically examined. Skinks are diverse and abundant in Australia—more diverse than anywhere else in the world [62], with 470 described species [63] that coexist with many of the world’s most venomous snakes (large Australian elapids). Previously, it has been shown that some species of Australian skinks are resistant to the blood-acting toxins of sympatric venomous snakes [64]. In addition, simple survival assays showed that some Australian skinks (*Anomalopus leuckartii*, *Ctenotus robustus*, *Egernia cunninghami*, *Egernia striolata*, and *Liopholis whitii*) were able to withstand high venom doses of *Acanthophis* (death adders), *Notechis* (tiger snakes), and *Pseudonaja* (brown snakes) venom [65]. This leads to the testable hypothesis that the strong presence of neurotoxic snakes in Australian ecosystems creates a potent selective pressure that could drive the evolution of toxin resistance in skinks, potentially leading to multiple independent adaptive events given the skinks’ wide radiation.

As such, this study aimed to: (i) determine if Australian skinks have evolved resistance to snake α-neurotoxins via modifications of the nAChR α1 subunit; (ii) identify the specific amino acid changes responsible and assess whether they represent convergent evolution with other taxa (e.g., the R187 and N-glycosylation motifs known from mammals); (iii) evaluate how many times such adaptations have arisen independently across the skink phylogeny; and (iv) explore bioactivity using the death adder (*Acanthophis wellsi*) venom, which has been previously shown to be a broad-acting model alpha-neurotoxic Australian elapid species [26], with death adders in general being models for alpha-neurotoxicity due to their venom being dominated (>90–95%) by alpha-neurotoxins [66,67]. We also sought to place these findings in evolutionary and ecological contexts, testing whether the occurrence of resistance-related mutations correlates with a skink’s exposure to venomous snakes. The significance of this study is that uncovering venom resistance in skinks would extend our understanding of how widespread and repetitive this adaptive phenomenon is in nature. It would highlight the skinks as an additional model for studying protein evolution under toxin pressure, complementing known mammalian and amphibian cases. Ultimately, this research sheds light on the adaptive significance of venom resistance, illustrating how molecular evolution enables certain species to survive in otherwise lethal ecological interactions.

## 2. Results

### 2.1. Convergent Molecular Adaptations in Skink nAChR α1 Subunits

**Figure 1:**
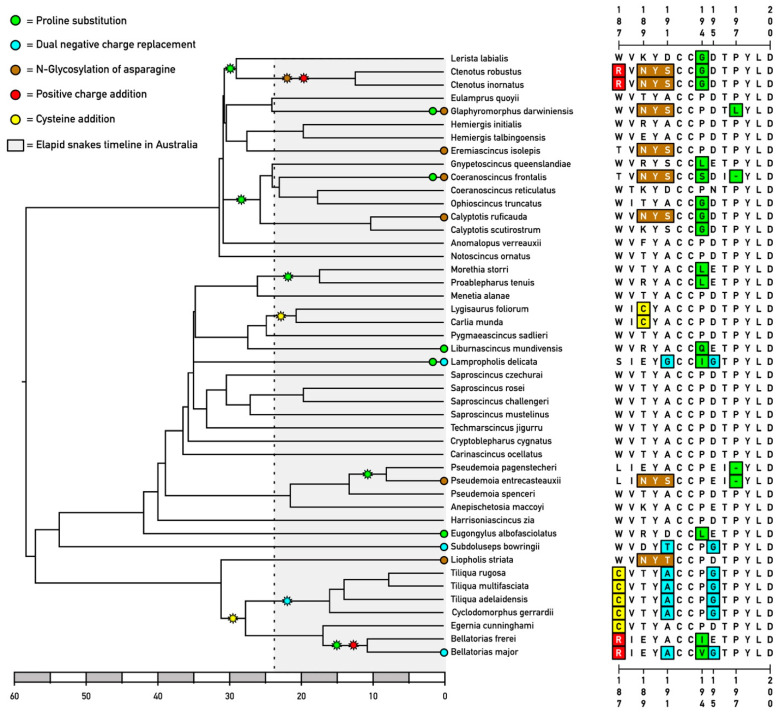
Australian skink orthosteric site mutations linked to α-neurotoxin resistance mapped on the organismal phylogeny, with a scale bar representing millions of years of diversification. The initial tree was obtained from Timetree.org [69] and has been adapted to include data from the key literature [70,71,72,73,74,75,76,77,78,79,80,81]. Phylogenetic ambiguities at internal nodes are represented as soft polytomies. Character history was traced through Maximum Parsimony-based ancestral sequence reconstruction executed in Mesquite v.3.81 [82]. Shading shows the approximate timing (~24 MYA) of the arrival of elapid snakes in Australia [68].

An analysis of skink nAChR sequences revealed diverse mutations at the toxin-binding site. Mapping these mutations on the Australian skink phylogenetic tree revealed that there are twenty-five resistance motifs scattered across unrelated clades, rather than clustered in a single lineage, with twelve of these lineages containing multiple evolutions of resistance motifs (Figure 1). These genera are separated by substantial evolutionary distances, indicating that the traits did not arise from a common modified ancestor but rather evolved in convergently. The timing of the evolution of venom resistance motifs is subsequent to the arrival of elapid snakes into Australia (Figure 1) [68], with the higher-level presence of resistance motifs suggestive of some resistance motifs evolving in lineages subjected to elapid snake pressure prior to their dispersal into Australia.

**Figure 2:**
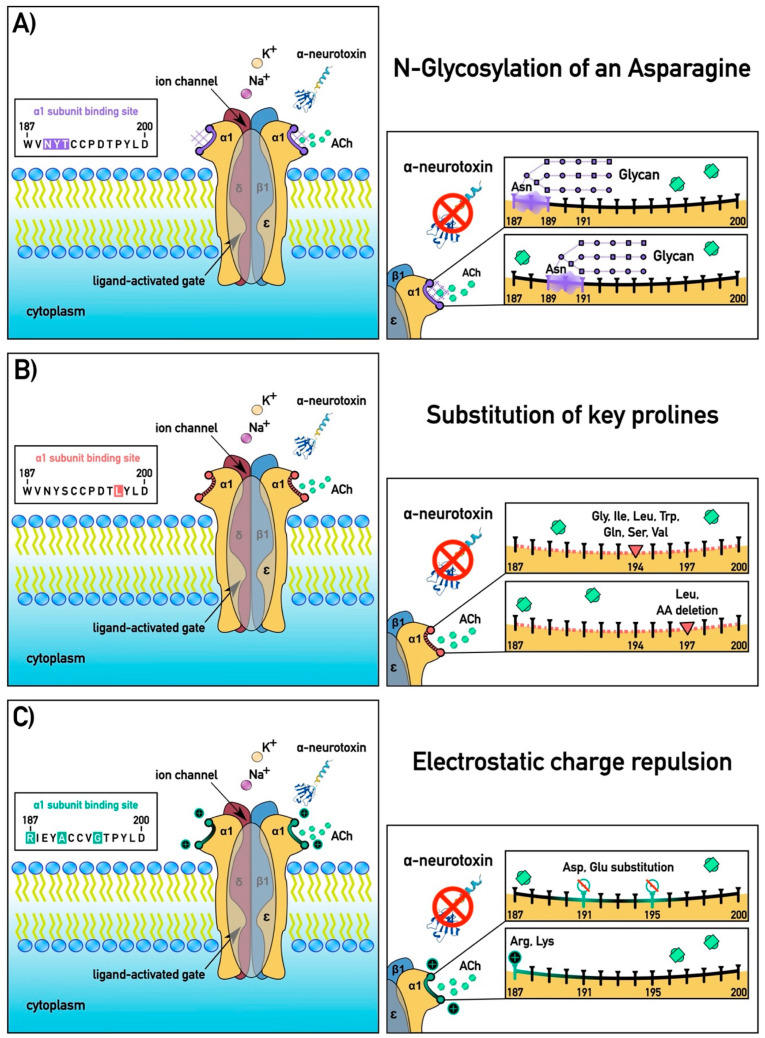
The three key resistance mutations documented as evolving in Australian skinks: (**A**) N-glycosylation of arginine at position 189, with the bulky glycan chains sterically interfering with α-neurotoxin docking; (**B**) substitution of prolines 194 or 197, thereby changing the orthosteric site secondary structure, leading to steric hindrance of α-neurotoxin docking; or (**C**) changing the electrostatic state of the orthosteric site either through the introduction of a positive charge at position 187, resulting in a same-charge repulsion of the positively charged α-neurotoxins, or the loss of negative charges at the position, resulting in decreased affinity for the α-neurotoxins.

Evolution of steric hindrance motifs occurred on nineteen occasions, consisting of N-glycosylation, proline substitutions, and the introduction of novel cysteines (Figure 2). The N-glycosylation form of steric hindrance resistance mutations all evolved at position 189 on seven occasions: (1) in *Calyptotis ruficauda*, (2) in *Coeranoscincus frontalis*, (3) in the last common ancestor (LCA) of *Ctenotus*, (4) in *Eremiascincus isolepis*, (5) in *Glaphyromorphus darwiniensi*, (6) in *Liopholis striata*, and (7) in *Pseudomoia entrecasteauxii.* Steric hindrance mutations in the form of proline substitutions, which would change the three-dimensional architecture of the domain due to the loss of this secondary structure “kinking” amino acid and lower (but not abolish) toxin binding, occurred on a total of ten occasions. At position 194, this was observed as evolving on seven convergent occasions: (1) in the *Bellatorias* LCA; (2) in the LCA of *Calyptotis, Coeranoscincus*, *Gnypetoscincus*, and *Ophioscincus* (with this secondarily lost in *Coeranoscincus reticulatus*); (3) in the LCA of *Ctenotus* and *Lerista*; (4) in the LCA of *Morethia* and *Proablepharus*; (5) in *Eugongylus albofasciolatus*; (6) in *Lampropholis delicata*; (7) and in *Liburnascincus mundivensis*. Proline substitution at site 197 occurred on one occasion, in *Glaphyromorphus darwiniensis*. Rather than a substitution, the deletion of the proline codon has convergently evolved on two occasions at position 197: in *Coeranoscincus frontalis*, and again in the LCA of *Pseudemoia*. The introduction of a novel cysteine, which would change the three-dimensional structure of the binding pocket—thereby impeding toxin binding—was shown to have convergently evolved two times: at position 187 in the LCA of *Bellatorias*, *Cyclodomorphus*, *Egernia*, and *Tiliqua*, with this mutation subsequently replaced by R187 in *Bellatorias*, and at position 189 in the LCA of *Carlia* and *Lygisaurus*.

In addition to steric hindrance, we also retrieved six instances of the evolution of motifs linked to α-neurotoxin resistance by changes in the electrical state of the orthosteric site. The R187 electrostatic mutation occurred on two occasions: in the *Ctenotus* LCA and in the *Bellatorias* LCA. Most skink species retain a negatively charged amino acid at either position 191 or 195 (typically 195). However, previous research has shown that the simultaneous loss of negatively charged amino acids at positions 191 and 195 by uncharged amino acids at positions 191 and 195 significantly reduces the susceptibility to neurotoxins [54]. This trait was observed evolving on four convergent occasions: in the LCA of *Cyclodomorphus* and *Tiliqua*, in *Bellatorias major*, in *Lampropholis delicata*, and in *Subdoluseps bowringii*.

### 2.2. Comparison with Other Vertebrates

Alignment with known resistant sequences: Aligning skink nAChR sequences with those of other venom-resistant vertebrates revealed remarkable similarity at the molecular level. The mutation to encode for an arginine (R) in skinks occurs at the exact position (187) as in Honey Badgers and hedgehogs [50], as well as caecilian amphibians [55], demonstrating an identical evolutionary solution in amphibians, lizards, and mammals separated by >300 million years of evolution. The introduced N-glycosylation sites in skinks occupy the same 189 position of the receptor as the glycosylation in elapid snakes such as cobras [27,57]. In contrast, mongooses have their N-glycoslation at a different but closely positioned site (187) [3]—the position where *Ctenotus* and *Bellatorias* have evolved their R mutation. Caecilian amphibians have had N-glycosylation mutations sequenced in at either 187 or 189 (but not both in a single species) [55]. This suggests there are “hot spot” sites in the receptor where resistance is conferred by particular mutations.

### 2.3. Signs of Selection Analyses Reveal Complex Evolutionary History with Instances of Positive Selection

The “one-ratio” model we tested in codeml (Model = 0; NSSites = 0) found a global ω of 0.52, which is associated with purifying selection. The “one-ratio” model we tested in codeml (Model = 0; NSSites = 0) found a global ω of 0.52, which is associated with purifying selection. However, site models tell us more about the complex evolutionary history of the orthosteric site of the alpha-1 subunit of the nAchR. Despite the protein-coding region evolving under an overall conservative tendency, the likelihood ratio test comparing the likelihood scores of site models M7 (beta) and M8 (beta + ω > 1) yielded a test statistic of LRT = 40.34, which is highly significant under a chi-squared distribution with 2 degrees of freedom (critical value = 13.82, *p* < 0.001). This result strongly supports model M8 as a better fit to the data, indicating the presence of positively selected sites. This statement is confirmed by the BEB output, which flagged site 189 under strong positive selection (*p* = 100%). Site 189 harbours the mutation in which an asparagine (N) is introduced, creating an instance of glycosylation (NXS/T motif at sites 189–191), which results in the formation of a shrubbery-like structure that sterically prevents toxins from binding to the receptor. Moreover, for N-glycosylation, site 189 also displayed a novel cysteine in the LCA of genera *Carlia* and *Lygisaurus*, which creates a structural kink that is supposed to decrease toxin binding due to a tertiary structure modification of the entire alpha-1 subunit.

### 2.4. Receptor Binding Assays

**Figure 3:**
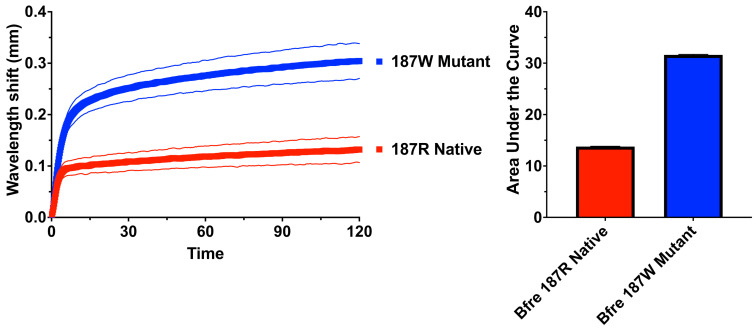
Biolayer interferometry assaying of native and mutant *Bellatorias frerei* mimotopes to test if the arginine at position 187 that confers resistance in the Honey Badger (*Mellivora capensis*) [53] also confers resistance in skinks. The cysteine doublet was replaced by a serine doublet during peptide synthesis to prevent uncontrolled postsynthetic thiol oxidation [83]. Venom used for binding test was *Acanthophis wellsi*. Values are N = 3 mean with standard deviations shown for both the line graph and the area under the curve bar graphs.

Testing of the native and mutant versions of *Bellatorias frerei* confirmed the importance of the arginine mutation at position 187 (Figure 3). The venom tested was *Acanthophis wellsi*, which is dramatically the most potent of all death adders [26]. The in vitro binding experiments provided direct evidence of the protective effect of the 187R resistance motif, previously characterised as responsible for the resistance to cobra venom by the Honey Badger (*Mellivora capensis*) [53]. The native (resistance motif) version was bound much less strongly than the mutant (arginine replaced by the tryptophan found in non-resistant species), which was bound with a 130% increase relative to the native form. This is notable, as *A*. *wellsi* has been previously shown to overcome other types of resistance motifs found in Australian dwarf varanid lizards.

## 3. Discussion

The evolutionary arms race of increased toxin and subsequent toxin resistance in prey and predators is a critical adaptation and coevolutionary dance played out in several mammalian taxa but was unexplored in skinks, a major radiation of lizards in Australia. We explored this interaction in Australian skinks and found that Australian skinks have independently evolved molecular solutions to the threat of snake venom that closely mirror those found in vastly different animals. We show that there have been multiple independent origins of α-neurotoxin resistance across diverse skink lineages. The results in this study are congruent with the theory that there are “hot spot” sites in the receptor where any suitable mutation can yield a resistance effect [36], and evolution has repeatedly hit these spots in different taxa. This is a striking case of convergent evolution between lizards, snakes, and mammals via a complex trait (post-translational modification addition). The overall pattern of molecular evolution in the alpha-1 subunit of the nicotinic acetylcholine receptor (nAchR) reflects a strong influence of purifying selection, as evidenced by the global ω value of 0.52 obtained under the one-ratio model in codeml. This finding is consistent with the well-established functional constraints on this receptor, which plays a critical role in the highly conserved neuromuscular signalling pathway. The preservation of function is paramount, as the receptor’s interaction with acetylcholine is essential for muscle contraction across vertebrates. Consequently, widespread adaptive change across the orthosteric site would likely disrupt this fundamental role and be selected against.

Despite this global signal of constraint, our site-specific models reveal a more nuanced evolutionary dynamic. A significant likelihood ratio test (LRT = 40.34, *p* < 0.001) between site models M7 and M8 strongly supports the presence of positive selection at discrete sites within the receptor. Among these, site 189 emerged as a clear outlier, flagged with high posterior probability (BEB *p* = 100%) for undergoing diversifying selection. This site is of particular interest because it serves as a hotspot for functionally convergent adaptations across multiple reptilian lineages. The introduction of an asparagine at site 189 establishes a canonical N-linked glycosylation motif (N-X-S/T) spanning residues 189–191, leading to the addition of a sterically bulky glycan moiety. This glycosylation effectively creates a structural barrier that impedes α-neurotoxin binding, conferring resistance to snake venom—a striking example of convergent molecular evolution in response to shared ecological pressure.

Moreover, the evolution of an additional cysteine at this site in the last common ancestor (LCA) of the genera *Carlia* and *Lygisaurus* suggests an alternative or complementary resistance mechanism. This cysteine likely introduces a local disulfide bond or structural kink, further altering the receptor’s tertiary conformation in a way that diminishes neurotoxin affinity. These structural innovations demonstrate the flexibility of evolution in fine-tuning receptor function through post-translational modifications and microstructural rearrangements while preserving the core cholinergic functionality. Together, these findings illustrate a classic evolutionary compromise: while the nAchR must remain tightly conserved to fulfil its physiological role, selective pressure imposed by predator-derived α-neurotoxins has repeatedly targeted a narrow set of mutable positions. These “evolutionary hotspots” allow for the emergence of resistance without broadly compromising receptor function. Such patterns of constrained but targeted adaptation underscore the complex interplay between natural selection and structural constraint in the evolution of critical neural proteins.

Within Australian skinks, we uncovered twenty-five instances of the evolution of known resistance motifs [3,4,26,27,36,45,46,47,48,49,50,51,52,54,55,56,57,58] evolved. This reflects a widespread evolutionary response across the family in response to the invasion of the Australian continent by elapid snakes ~24 million years ago [68], resulting in an enormous selection pressure by these novel predators against previously biochemically naive prey. As some lineages contained multiple resistance motifs, there were, overall, thirteen lineages that contained at least one resistance motif: (1) substitution of proline at position 194 in the LCA of *Ctenotus* and *Lerista*; (2) substitution of proline occurred at position 197 in *Glaphyromorphus darwiniensis*; (3) N-glycosylation at position 189 in *Eremiascincus isolepis*; (4) substitution of proline at position 194 in the LCA of *Calyptotis, Coeranoscincus, Gnypetoscincus,* and *Ophioscincus* (with this secondarily lost in *Coeranoscincus reticulatus*); (5) substitution of proline at position 194 in the LCA of *Morethia* and *Proablepharus*; (6) novel cysteine at position 189 in the LCA of *Carlia* and *Lygisaurus*; (7) substitution of proline at position 194 in *Liburnascincus mundivensis*; (8) loss of both negatively charged amino acids (positions 191 and 195) in *Lampropholis delicata*; (9) deletion of the proline codon at position 194 in *Pseudemoia*; (10) substitution of proline at position 194 in *Eugongylus albofasciolatus*; (11) loss of both negatively charged amino acids (positions 191 and 195) in *Subdoluseps bowringii*; (12) N-glycosylation at position 189 in *Liopholis striata*; and (13) novel cysteine at position 187 in the common ancestor of *Bellatorias*, *Cyclodomorphus*, *Egernia*, and *Tiliqua*. The twelve additional mutations that evolved within these lineages were as follows. The R187 mutation occurred on two occasions: (1) in the *Ctenotus* LCA, and (2) in the *Bellatorias* LCA. N-glycosylation evolved at position 189 on five occasions: (1) in *Calyptotis ruficauda*, (2) in *Coeranoscincus frontalis*, (3) in *Ctenotus* LCA, (4) in *Glaphyromorphus darwiniensis*, and (5) in *Pseudomoia entrecasteauxii*. A substitution of proline at position 194 occurred on two convergent occasions: (1) in *Bellatorias* LCA, and (2) in *Lampropholis delicata*. Proline codon deletion at position 197 occurred once, in *Coeranoscincus frontalis*. The loss of both negatively charged amino acids (positions 191 and 195) occurred on two occasions: (1) in the LCA of *Cyclodomorphus* and *Tiliqua*, and (2) in *Bellatorias major*. (*Anomalopus leuckartii*, *Ctenotus robustus*, *Egernia cunninghami, Egernia striolata*, and *Liopholis whitii*) were able to withstand high doses of *Acanthophis* (death adders), *Notechis* (tiger snakes), and *Pseudonaja* (brown snakes) venom [65]. It was notable that *Anomalopus verreauxii* sequencing did not reveal any resistance motifs, when the close relative *A*. *leuckartii* was shown previously by *in vivo* testing to be resistant to snake venom [65]. This underscores what a dynamic trait venom resistance is.

These multiple origins of venom resistance likely correspond to separate coevolutionary “hotspots”—for example, each time skink niches were colonised by neurotoxic elapid snakes, selection drove the local skink population toward resistance. Over evolutionary time, this led to a mosaic of resistant lineages. The convergence of which mutations evolved (often the sites at which arginines or N-glycosylation were introduced) and at the same sites (most mutations occurring in the aromatic binding site at positions 189 and 191 [36], or the proline binding site at 194 and 197 [36]), indicates that there are limited effective evolutionary solutions available, and evolution finds those solutions repeatedly when needed. Our findings show that skinks have embraced diverse resistance strategies in different contexts. This suggests that skinks, despite their smaller size and different ecology, have explored a similar solution space of molecular changes as other venom-resistant species.

Our study demonstrates that Australian skinks have independently evolved molecular solutions to the threat of snake venom that closely mirror those found in vastly different animals [3,4,26,27,36,45,46,47,48,49,50,51,52,54,55,56,57,58]. The recurrence of arginine (R) substitutions and N-glycosylation motifs at the nAChR orthosteric site across amphibians, mammals, and reptiles is a striking example of convergent evolution. It highlights how a specific molecular target (the orthosteric site of the nAChR α1 subunit) and specific sites (particularly the aromatic site at positions 187 and 189 [36], and the protein site at positions 194 and 197 [36]) on that target represent evolutionary “weak points” that natural selection repeatedly acts upon to confer an adaptive advantage. The fact that skinks (ectothermic reptiles that are prey to neurotoxic elapid snakes), caecilian (ectothermic amphibians that are prey to neurotoxic elapid snakes) and Honey Badgers (endothermic mammals that are predators of neurotoxic elapid snakes) ended up with an identical receptor mutation (R187) underscores the predictability of evolution under strong selective pressures. The R187 effect was validated here in a reptilian context, confirming our previous work on the Honey Badger mammalian version that this substitution impedes toxin binding [53]. Similarly, the deletion of the codon encoding for the proline at position 197 had only been previously documented in certain Afro–Asian venom-resistant terrestrial pythons [46,53], with the discovery in skinks representing another case of convergent evolution for resistance motifs at the same molecular hotspots.

Based on the current sequencing results, thirteen Australian skink lineages with at least one α-neurotoxin resistance motif, with twelve more evolutions of resistance motifs within these lineages, results in at least twenty-five total times these venom resistance motifs have evolved. This is comparable to what has been documented for other lineages such as caecilians, mammals, and varanid lizards.

Currently, caecilian amphibians show at least sixteen lineages with α-neurotoxin resistance, with some lineages evolving multiple forms of resistance for a total of twenty-two times resistance to α-neurotoxic venoms has evolved [55]. Steric hindrance has evolved on twenty occasions within caecilians: N-glycyosylated asparagines evolved on eight occasions (four times at position 187 and four at position 189); proline substitutions evolved on ten occasions (nine times at position 194 and one at position 197); novel cysteines evolved on two occasions (one time at position 187 and again at position 189). Electrostatic charge repulsion also evolved within caecilians, with R187 evolving on two independent occasions within these amphibians.

Mammals—which may be predators or prey of neurotoxic snakes, depending on the relative sizes on either side of the interaction—have at least seventeen lineages that have evolved α-neurotoxin resistance motifs, with several lineages evolving multiple forms of resistance motifs for a total of twenty evolutions of venom resistance [51]. Steric hindrance has evolved at least nine times within mammals, consisting of N-glycosylations, proline substitutions, and novel cysteines. Only the last common ancestor of the Herpestidae (mongooses and meerkats) evolved the N-glycosylation at position 187 form of steric hindrance within mammals. The proline substitution mechanism of steric hindrance evolved on six occasions: five times at position 194 and once at position 197 (with the evolution at 197 occurring simultaneously with a 194 substitution within the Herpestidae). Novel cysteines evolved twice as a steric hindrance mechanism: on one occasion at position 187 and again at position 189. Electrostatic repulsion evolved on at least eleven occasions within mammals. R187 convergently evolved nine times, and a novel K187 (which remains to be experimentally validated but is suggestive as imparting resistance due to lysine being a positively charged amino acid like arginine) evolved on two occasions: once in the genus *Genetta* (small African carnivores including the genet) and again in the African Thicket Rat (*Grammomys surdaster*; K187).

Varanid lizards show extensive diversification of resistance within this single lineage due to interactions both as predators and prey of neurotoxic elapid snakes [26,54]. At the base of this clade, resistance evolved in the form of the loss of both charged amino acids at positions 191 and 195. There was a subsequent loss of resistance in the aquatic lineages *V*. *indicus* and *V*. *salvator*, hypothesised to be due to lower levels of interaction with elapids, with the modification of the receptors subjected to a “use it or lose it” selection pressure. Other lineages also convergently evolved to a state of lower levels of resistance (but not loss) through the re-evolving of charged amino acids at position 191 (two times: *V*. *giganteus* and again in the LCA of *V*. *komodoensis* + *V*. *varius*). Within the “*Odatria*” subgenus, there were multiple losses and gains paralleling ecological niche diversification into arboreality (associated with losses congruent with the lack of arboreal elapid snakes in Australia) or burrowing (associated with heightened resistance levels) [26].

Evolving venom resistance is clearly beneficial for skinks that live alongside venomous snakes—it can mean the difference between life and death during a snake encounter. In the context of predator–prey interactions, our results highlight a case of prey evolving a defence to a predator’s primary weapon (neurotoxic venom). For skinks, the payoff of resistance is immediate: surviving a snake bite allows them to escape, heal, and potentially reproduce, so even partial resistance could be strongly selected for. We see evidence of this in how tightly correlated the presence of resistance mutations is with snake exposure in skinks, rapidly evolving on a myriad of occasions subsequent to the invasion of Australia by elapid snakes ~24 million years ago [68].

By comparing skinks to other venom-resistant animals, we can draw broader conclusions about this evolutionary phenomenon. Mammals like mongooses and Honey Badgers evolved resistance in the context of predation on snakes—i.e., as predators that could afford to attack venomous snakes once immune to their venom [45,50,51]. Skinks, conversely, evolved it as prey to avoid being killed. Despite these differences, the convergent molecular outcomes suggest that whether for offense or defence, the evolutionary pressure yields similar adaptations. Caecilian amphibians, lizards, and snakes add to the tapestry of taxa with modified nAChRs [26,27,47,55]. Birds that eat elapid snakes, interestingly, have not shown such modifications, which is hypothesized to be due to having different predation strategies and mechanical resistance conferred by the thick leg scales of the birds [27]. This makes the lizard–mammal convergence even more remarkable. Our findings with skinks thus fill an important gap: they show that squamate reptiles as prey (beyond just a few known cases) participate in this convergent trend, bridging what was previously a mammalian predator-and-snake prey-dominated narrative. In the big picture, across vertebrates, whenever a niche or survival challenge involves elapid-like neurotoxins, evolution tends to tweak the same receptor in similar ways—a testament to both the potency of these toxins and the ingenuity of natural selection.

The results from Australian skinks can be viewed through the lens of coevolutionary theory. We have a clear agent of selection (snake venom) and a target of selection (the orthosteric site of the skink nAChR’s α1 subunit). The repeated emergence of resistance in skinks could in turn influence snake evolution, leading to an ongoing arms race. For instance, there is a reciprocal selection pressure for snakes to evolve altered venom—possibly increasing the concentration of α-neurotoxins, evolving toxins with different targets, or adding components that circumvent the skink’s resistance (such as cytotoxins or digestive enzymes that cause harm even if neurotoxins fail). There is a precedent for such counter-adaptations, as seen in Australian varanid lizards, where the venom of the death adder Acanthophis rugosus circumvents the resistance displayed by the varanid clade consisting of the sympatric species *Varanus acanthurus*, *V*. *insulanicus*, *V*. *ocreatus*, *V*. *primordius*, and *Varanus storri*, species that were highly resistant to the venom of other species of death adder [26].

In Australia, where venomous snakes are abundant and skinks are a major prey group, this interplay could therefore be a significant driver of venom evolution. Our study sets the stage for testing such hypotheses by identifying which skink species are resistant; future work could examine if neurotoxic snakes preying on those species have any differences in venom compared to those that prey on non-resistant prey. While comprehensive, our study has some limitations. We inferred resistance from molecular and in vitro data rather than measuring survival in wild snakebite scenarios. There may be other factors contributing to venom resistance (such as circulating anti-toxins in blood) that we did not investigate that may contribute to venom resistance, with this current study focusing solely on a particular receptor mechanism. Additionally, our phylogenetic sampling, while broad, did not include every Australian skink lineage; additional sampling may reveal instances of known resistance motifs or even other novel mechanisms. Despite these caveats, the consistency of our findings across multiple independent lineages gives us confidence in the general conclusions.

Building on this work, future studies could explore genome-wide adaptations in venom-resistant skinks to see if other genes show coevolution, such as those related to blood clotting, in light of prior work showing that Australian skinks are also resistant to blood-acting venoms [64]. Functional studies using gene editing (e.g., CRISPR to introduce skink-like mutations into model organisms) could directly demonstrate increased survival after venom exposure, further cementing causality. Another avenue is to study the population genetics of these resistance traits: do they show signs of selective sweeps in skink populations? Or are there polymorphisms in areas where snake pressure is variable? Finally, investigating other reptile groups whether as prey (geckos) or as predators (non-elapid snakes such as the *Aspidites* species Black-headed Python and Woma) for similar receptor modifications would help determine why some lineages evolve this adaptation and others do not, providing a fuller picture of the evolutionary playbook against venoms. Future studies may also include cells (such as oocytes) artificially expressing full receptors that have orthosteric sites corresponding to native and mutated skink orthosteric sites to further investigate the physiological impact of the variations revealed in this study. Such studies will be particularly important to investigate the impact proline substitutions have upon the secondary structures of the receptor pockets.

By unravelling how skinks dodge death by venom, we gain insights that could inform fields as diverse as evolutionary biology, ecology, and even biomedical approaches to treating snakebite envenomation. In essence, the evolution of extensive venom resistance in Australian skinks stands as a testament to nature’s ingenuity. Faced with the threat of rapid, venom-induced paralysis, these little reptiles evolved molecular armor at the synapse. The evolution of venom resistance might have opened up new ecological niches for skinks. If a skink species can survive in areas teeming with deadly snakes, it might exploit resources (food, habitat) that other reptiles or rodents who lack such resistance cannot, potentially reducing competition. This could partly explain the success and abundance of certain skink lineages in Australia’s harsh, predator-rich environments. In essence, venom resistance could be a critical adaptive trait that has shaped skink evolutionary history and enabled them to thrive alongside venomous fauna.

This study provides the first comprehensive evidence that venom resistance is widespread in Australian skinks, driven by convergent molecular adaptations in the nicotinic acetylcholine receptor. We identified key mutations that recur across multiple skink lineages, effectively blocking the action of deadly snake neurotoxins. These mutations closely parallel those found in other venom-resistant mammals and reptiles, both in location within the orthosteric site and type of mutation, underlining a common evolutionary strategy. The independent evolution of similar resistance in so many skinks highlights the intense selective pressure exerted by venomous snakes in Australia since their arrival and the limited but repeatable set of solutions that evolution can employ to overcome this challenge. This convergence across species and even classes of vertebrates is a powerful demonstration of how predictable evolution can be when constrained by biochemical targets and functional needs. Our findings not only illuminate an intriguing aspect of skink biology and Australian food-web dynamics but also contribute to the broader understanding of coevolution. They reveal how a specific protein in the neuromuscular system becomes a battleground in the evolutionary war between predator and prey. This provides a foundation for future work, which may include receptor expression assays, behavioural studies, and toxin challenge experiments.

## 4. Materials and Methods

### 4.1. Sample Collection and Species Selection

We obtained museum tissue samples from a preserved broad range of Australian skinks, covering all major taxonomical clades and ecological niche occupations within the family Scincidae on the continent. No live animals were worked with for this study. Tissue work was undertaken under University of Queensland Animal Ethics Approval 2021/AE000075. Our sample included 45 species (spanning 35 genera) representing diverse ecologies and varying degrees of potential snake exposure. All samples were sourced non-lethally from museum specimens to maximise taxonomic breadth without harming wild populations.

### 4.2. DNA Extraction and nAChR α1 Gene Sequencing

Target gene: We focused on the gene encoding the muscle nicotinic acetylcholine receptor alpha-1 subunit (CHRNA1). This subunit contains the principal component of the orthosteric binding site for α-neurotoxins and is known to harbour resistance-conferring mutations in other taxa [3,4,26,27,36,45,46,47,48,49,50,51,52,54,55,56,57,58].

Molecular methods: Genomic DNA was isolated using the DNeasy Blood & Tissue kit (QIAGEN, Carlsbad, CA, USA) using an optimised spin column-based protocol according to the manufacturer’s instructions. Prior to the DNA extraction, the tissues were rinsed with 10% of phosphate-buffered saline (PBS) to remove the 70% ethanol preservative. Homogenised tissue samples (25 mg) were mixed with Proteinase K lysis solution and 56 °C shake-incubated for 3 h. Centrifugation steps were undertaken followed by washing with wash buffer solutions. Post-elution, the DNA concentration and purity were determined using the Nanodrop 2000 UV–Vis Spectrophotometer (Thermo Fisher Scientific, Waltham, MA, USA). The isolated genomic DNA was stored at −20 °C until it was used for PCR amplification. A ~200 base pair range corresponding to chrna1 (muscular nAChR gene) was amplified by the following locus-specific primer-directed PCR protocol:

- Samples were amplified using following primers designed for the nAChR orthosteric using our *Tiliqua rugosa* genomic sequence:
  ○ Forward 5′ TGAGTAACTTCATGGAGAGCGG 3′.
  ○ Reverse 5′ TGTGGGCAGATAAAACACTAAGCC 3′.
- PCR reaction contents were as follows:
  ○ 25 μL of Taq PCR master mix.
  ○ 3 μL of each primer (10 μM).
  ○ 500 ng of DNA.
  ○ PCR water to adjust to the 50 μL total PCR reaction volume.
- The PCR reaction conditions were as follows:
  ○ Initial denaturation at 95 °C for 3 min (all subsequent denaturation steps were at 95 °C for 30 s).
  ○ Annealing was at 55 °C for 30 s.
  ○ Extension was at 72 °C for 1 min.
  ○ The PCR steps of denaturation, annealing, and extension were repeated for 35 cycles.
  ○ Final extension at 72 °C for 10 min.

Sequencing of the primer-directed locus-specific amplified PCR products was undertaken at the Australian Genome Research Facility, University of Queensland, Australia, using the automated dideoxy sequencing method dual-direction sequencing.

### 4.3. Identification of Putative Resistance Mutations

The sequence reads were aligned and manually curated using the Aliview v.1.1 software (alignment viewer and editor) and Expasy (translate tool) to ascertain the relative presence of known resistance motifs [3,4,26,27,36,45,46,47,48,49,50,51,52,54,55,56,57,58] in the ligand-binding domain of the α1 subunit of the nAChR. To detect N-glycosylation motifs, we screened the aligned sequences for the presence of the N-linked glycosylation motifs N-X-S/T, where X ≠ cysteine (C) or proline (P), and S/T indicates either serine (S) or threonine (T) as the last part of the amino acid triplet [59,60,61]. Any introduction of an asparagine followed two positions later by a serine or threonine at sites corresponding to the toxin-binding interface was noted as a candidate resistance mutation.

### 4.4. Data Analysis and Ancestral State Reconstruction

To provide a framework for adaptive selection analyses and trait mapping, we employed a CHRNA1 gene phylogeny adapted from a dated representative organismal phylogeny available from Timetree.org [69]. We applied a codon-based approach to test for positive selection (adaptive evolution) using the plug-in codeml available in PAML v4.10.1 [84]. Our goal was to investigate if the toxin-binding region would show dN/dS ratios significantly > 1, indicating diversifying selection likely driven by venom interaction. To do so, we applied the so-called “one-ratio” model and two site models in tandem (M8 vs. M7) available in codeml. The one-ratio model aims to identify patterns of adaptive evolution across the entire phylogeny by calculating a global, representative selection coefficient ω (=dN/dS) indicating the evolutionary direction of the analysed region across the featured taxa. The one-ratio model is specified in codeml by setting the model to 0 and NSSites to 0. Site models are aimed at identifying which individual codons are under positive selection. Such analyses consider the selection coefficient ω constant across the tree while allowing for the selection coefficient to vary across single sites. We opted for site-based codon substitution models M7 (beta) (Model = 0, NSSites = 7) and M8 (beta and ω > 1) (Model = 0, NSSites = 8). Models M7 and M8 were both tested, as they depict alternative evolutionary scenarios that make them frequently compared in adaptive evolution studies. M8 allows for the selection coefficient ω to exceed 1, hypothesising that individual sites might be under positive selection. M7, on the other hand, constrains ω between 0 and 1, providing a null hypothesis-like scenario to test against. A likelihood ratio test (LRT) at degrees of freedom (hereafter, d.f.) = 2 and *p* < 0.001 (critical value = 13.816) offers the possibility to support or refute a scenario where positive selection fits the data. Sites under positive selection are detected by site models in codeml through the Bayes Empirical Bayes (BEB [85]. Sites were considered to be positively selected when the posterior probability yielded by the BEB assay was above 95%.

The presence or absence of key resistance-associated mutations (tandem negative charge substitution, glycosylation motif, etc.) was treated as discrete traits and mapped onto the phylogeny. For example, if two distantly related skinks both have an R187 mutation at the binding site, we assessed whether this arose convergently or via a common (deep) ancestor. We traced the evolutionary history of each resistance modification through the parsimony-based ancestral trait reconstruction available in Mesquite v.3.81 [82]. The evolution of traits was reconstructed under the ACCTRAN (Accelerated Transformation) criterion, the default option in Mesquite, which favours transformations occurring as close as possible to the root [86,87].

### 4.5. Receptor Binding Assays

#### 4.5.1. Mimotope Design and Preparation

- 14-amino-acid-long short peptide mimotope corresponding to the orthosteric site of the *Bellatorias frerei* skink muscle-type nAChR α-1 subunit was synthesised as per previous studies [2,44,52,53,54].
- Post-synthesis uncontrollable thiol oxidation was prevented by the synthetic peptides having a serine doublet in place of the cysteine-doublet as per previous work [83].
- Mimotopes were dissolved in 100% dimethyl sulfoxide (DMSO) followed by 1:10 dilution with double-deionised water in order to make 50 µg/mL working stocks.
- All prepared mimotope stock solutions were stored at −20 °C for future use.

#### 4.5.2. Biolayer Interferometry Assay (BLI)

- A validated Octet HTX biolayer interferometry assay was used to measure neurotoxin-receptor binding affinities as per previously published protocols, as were data acquisition, processing, and statistical analyses [2,26,32,33,34,35,44,46,52,54,88]. It must be noted that the mimotopes only approximate the orthosteric site and cannot capture full conformational or functional receptor dynamics. *Acanthophis wellsi* venom (received under UQ Animal Ethics Approval 15 March 2021/AE000075) was used, as it has been previously shown to be a model species for potent broadly acting alpha-neurotoxic Australian elapid snake venom [26]. Blank sensors were used as negative controls.

## Data Availability

The data presented in this study are available on request from the corresponding author.
